# Structural Heart Interventions in the Elderly Population

**DOI:** 10.14797/mdcvj.1242

**Published:** 2023-05-16

**Authors:** Devang S. Parikh

**Affiliations:** 1 DeBakey Heart & Vascular Associates, Houston Methodist Hospital, The Woodlands, Texas, US

**Keywords:** transcatheter aortic valve implantation, aortic stenosis, valvular heart disease

## Abstract

Aortic stenosis is the most common valvular heart disease in the elderly population. Since the advent of transcatheter aortic valve implantation (TAVI) in 2002, the clinical indications for this alternative to a surgical replacement have continually expanded. While the treatment of octo- and nonagenarians can present significant challenges, here we present a case of TAVI in an elderly patient. Given her suitable anatomy and active lifestyle that had been limited by her disease state, the patient successfully underwent TAVI 3 weeks later and was discharged post-operative day 1. This case is the basis for providing five key points to remember about the work-up for TAVI for severe aortic stenosis in the elderly population.

Aortic stenosis (AS) is the most common valvular heart disease in the elderly population. This progressive condition results from thickening and calcification of the aortic valve (AV), which can lead to reduced functional capacity, increased hospitalizations, heart failure, and death when it becomes severe. In fact, when left untreated, the average survival is 50% at two years and 20% at five years from symptom onset. As of 2015, more than one in eight people over the age of 75 in the United States have moderate-to-severe AS.^1^ Current census data predicts that the number of nonagenarians is expected to quadruple to more than 8 million people in the United States by 2050, suggesting that the prevalence of AS may increase significantly.^2^

Since the advent of transcatheter aortic valve implantation (TAVI) in 2002 and based on high-quality randomized trial data, the clinical indications for this less invasive alternative to surgical aortic valve replacement (SAVR) have continually expanded to include high-, intermediate-, and low-risk surgical patients. While the treatment of octo- and nonagenarians can present significant challenges, here we present a case of TAVI in an elderly patient.

An 84-year-old woman presented to our valve clinic with dyspnea on exertion and a decline in her activity level over the past 6 months, which was particularly noticeable during her walks in the neighborhood. Her son also noted a change in her symptoms. Her other pertinent comorbidities included non-insulin dependent diabetes mellitus and hypertension. Initial workup by her primary cardiologist included an echocardiogram demonstrating a peak aortic velocity of 5.4 m/s, mean gradient of 68 mm Hg, and a calculated aortic valve area of 0.42 cm^2^, consistent with severe AS (Figure 1). Coronary angiography demonstrated mild diffuse coronary artery disease without any significant obstructive lesion.

**Figure 1:**
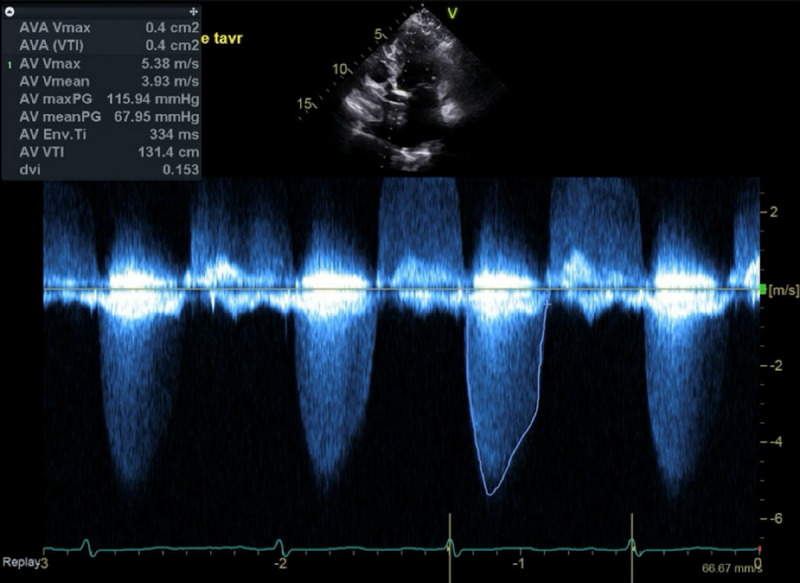
Continuous wave Doppler interrogation of aortic valve demonstrating mean gradient 68 mm Hg, Vmax 5.38 m/s, AVA 0.4cm^2^, DI 0.15 consistent with severe aortic stenosis.

We proceeded with a multidetector computed tomography scan of her chest, abdomen, and pelvis. Her annular dimensions showed an annular area of 307 mm^2^ and a perimeter of 63.3 mm (Figure 2). Her left iliac artery into her left common femoral artery measured 6 mm minimal in diameter (Figure 3). Her anatomy was suitable for TAVI with a 23-mm balloon expandable transcatheter heart valve. Her Society of Thoracic Surgery Predicted Risk of Mortality for SAVR was 2.5%. After discussion in a multidisciplinary heart team meeting to review her clinical condition and diagnostic testing, the patient successfully underwent TAVI 3 weeks later and was discharged on postoperative day 1.

**Figure 2:**
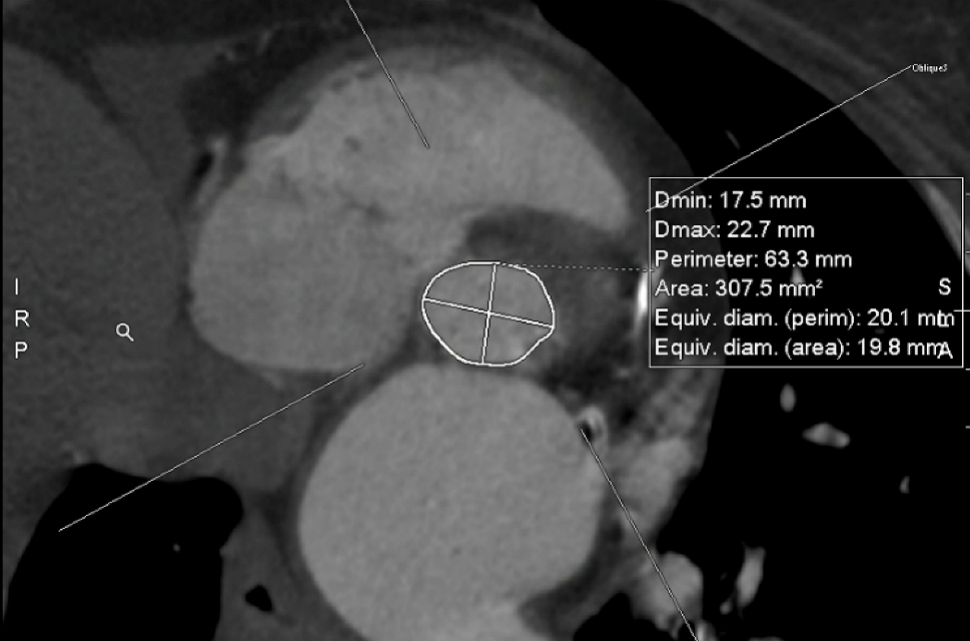
Annulus measurements for valve sizing with an annular area of 308 mm^2^ and a perimeter of 63.3 mm.

**Figure 3:**
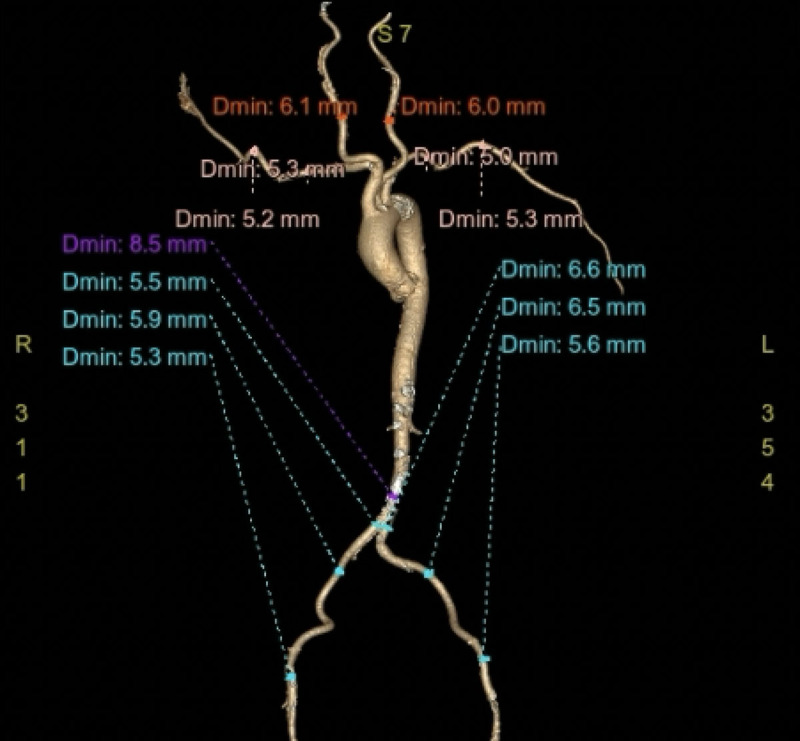
Minimal access site measurements of upper and lower extremities demonstrating a left iliofemoral system with minimal diameter 6 mm.

## Points to Remember

As demonstrated by the above case, key points to remember about the evaluation for TAVI for severe AS in the elderly population include the following:

- A multidisciplinary meeting including members from cardiology, cardiac surgery, cardiac imaging, and cardiac anesthesia is critical to a team-based approach toward decision making that can lead to improved outcomes for patients.^3^
- Numerous risk prediction tools, including Society of Thoracic Surgeons (STS) score and EuroScore II, can help guide clinical decision-making when considering structural interventions for elderly patients. Additionally, clinical status, anatomy, patient preferences, and frailty should be considered.^4^
- Multimodality imaging, including echocardiography, angiography, and multidetector computed tomography, is critical for pre-procedural planning.
- Some elderly patients may report mild or no symptoms with potentially life-threatening valvular disease. It is important to obtain a thorough history from patients and family members to illicit symptoms.
- Recent studies by Oh et al. and Mukunthan et al. demonstrated no significant difference in duration of hospital stay or in-hospital complications in patients 65-79 or ≥ 80 years of age undergoing TAVI.^5^,^6^
- Postprocedural care of elderly patients can be improved with the incorporation of a dedicated cardiac rehabilitation program individualized to patient abilities and pace.

## Competing Interests

The author has no competing interests to declare.
